# Clinical value of exogenous factor XIII for prolonged air leak following pulmonary lobectomy: a case control study

**DOI:** 10.1186/1471-2482-14-109

**Published:** 2014-12-15

**Authors:** Hidetoshi Inoue, Noritoshi Nishiyama, Shinjiro Mizuguchi, Koshi Nagano, Nobuhiro Izumi, Hiroaki Komatsu, Shigefumi Suehiro

**Affiliations:** Department of Cardiovascular Surgery, Osaka City University Graduate School of Medicine, 1-4-3 Asahi-machi, Abeno-ku, Osaka, 545-8585 Japan

**Keywords:** Pleural air leak, Postoperative care, Lung cancer surgery

## Abstract

**Background:**

We examined the effect of exogenous factor XIII (FXIII) concentrate in patients with prolonged air leak (PAL) after pulmonary lobectomy for non-small cell lung cancer.

**Methods:**

We performed a retrospective analysis of 297 patients who underwent pulmonary lobectomy between July 2007 and March 2014: 90 had an air leak on the first postoperative day, which resolved spontaneously within 5 days in 53 cases (SR group). FXIII concentrate was administered to the remaining 37 patients (PAL group) for 5 days. This group was subdivided into those in whom the air leak resolved during FXIII treatment (EF group) and those who needed additional intervention (inEF group). The clinical and perioperative characteristics of the groups were compared.

**Results:**

Although plasma FXIII activity did not differ significantly between the SR and PAL groups before surgery or on the fifth postoperative day, the proportional perioperative fall in FXIII activity was significantly greater in the SR group (33%) than the PAL group (22%, p = 0.044) and inEF group (14%, p = 0.048). On the fifth postoperative day, FXIII activity was significantly lower in the EF group than in the inEF group (74% *versus* 91%, p = 0.030). The optimal cut-off point for postoperative plasma FXIII activity to distinguish between the EF and inEF groups was 86%.

**Conclusions:**

Insufficient plasma FXIII consumption and lower postoperative FXIII activity may play a role in the resolution of PAL, and exogenous FXIII concentrate may be an effective, safe and non-invasive treatment.

## Background

Prolonged air leak (PAL) is a common complication of pulmonary resection, occurring in 8–26% of patients undergoing pulmonary lobectomy [1]. It is associated with prolonged hospital stay [2, 3] and other complications such as empyema [4]. Moreover, PAL occasionally requires further surgery, despite recent progress in the development of conservative treatments [1]. Older age, low body mass index (BMI) [5], poor wound-healing characteristics, low forced-expiratory volume (FEV) [6, 7], extensive pleural adhesions [6], and upper or bilobectomy [6, 8–10] reportedly increase the risk of PAL after lung resection. Diabetes mellitus, and low serum albumin and cholinesterase concentrations, all of which reflect impaired wound healing, might also be risk factors [11].

Pulmonary air leak normally resolves spontaneously if there is adequate chest drainage. If the pulmonary fistula persists, chemical pleurodesis may be undertaken with OK-432 (Picibanil®), tetracycline antibiotics, talc slurry, autologous blood or 50% glucose solution [12, 13]. The intrathoracic administration of diluted fibrin glue, a fibrinogen solution containing blood coagulation factor XIII (FXIII) and thrombin, has also been reported to be effective [14].

Blood coagulation factor XIII stabilizes fibrin clot and also plays a role in wound healing. In gastrointestinal surgery, administration of exogenous FXIII concentrate is reportedly effective for healing wounds and closing fistulae in patients with low perioperative plasma FXIII activity [15–17]. In thoracic surgery, exogenous FXIII concentrate has been used to treat persistent chylothorax [18]; however, little is known about the effect of FXIII when seeking to close a PAL.

The aim of this study was to examine the therapeutic effect of the systemic administration of exogenous FXIII concentrate for patients with PAL after pulmonary lobectomy for non-small-cell lung cancer, and to identify differences between patients who responded and who did not respond to treatment.

## Patients and methods

The medical records of 297 patients who underwent elective pulmonary lobectomy with mediastinal lymph node dissection for non-small-cell lung cancer at Osaka City University Hospital, Japan, between July 2009 and March 2014 were analyzed. Of these, 14 were excluded from the analysis because of incomplete data (five patients), the perioperative transfusion of blood or blood products (eight patients) and additional treatment for massive air leak within the first five perioperative days (one patient). Ultimately, the records of 283 patients were examined. No patients required postoperative mechanical ventilation or developed a bronchopleural fistula.

Surgery was performed through an axillary mini-thoracotomy (assisted by video) or posterolateral thoracotomy (when extended lobectomy, for example, chest wall resection, pulmonary arterioplasty or bronchoplasty, was necessary). During surgery, the presence of pulmonary air leak was tested by submerging the residual lung parenchyma in sterile saline solution and inflating it to a pressure of 15 cmH_2_O. Significant air leaks were addressed by additional suturing, applying polyglycolic acid sheets, or both. When considered necessary, fibrin glue was also sprayed on the lung surface to treat small alveolar air leaks. In all cases, a four lumen 24 French silicon chest tube was inserted once surgery was complete, and suction applied at −10 to −15 cmH_2_O until the next morning. On the first postoperative day, air leak and intrathoracic pressure were assessed by the surgeons, and suction was replaced by a water seal unless there was a massive air leak. Chest tubes were removed if there was no detectable air leak and less than 300 mL had been drained in the previous 24 h. When air leak persisted until the fifth postoperative day, 24 mL FXIII concentrate (Fibrogammin P®; CSL Behring, Tokyo, Japan) was administered intravenously once daily for 5 days. When an air leak still persisted after the 5-day course of FXIII concentrate had been completed, other methods of treatment such as pleurodesis, bronchial embolization or secondary operation were undertaken. Routine blood tests including plasma FXIII activity were measured before operation and on the fifth postoperative day. Plasma factor XIII activity was measured by means of a synthesized substrate assay, for which the normal range is considered to be 70–140% [19]. Change ratio in postoperative plasma Factor XIII activity were calculated by subtracting plasma FXIII activity on the fifth postoperative day from that measured before surgery, divided by the preoperative FXIII activity for each patient.

**Figure 1:**
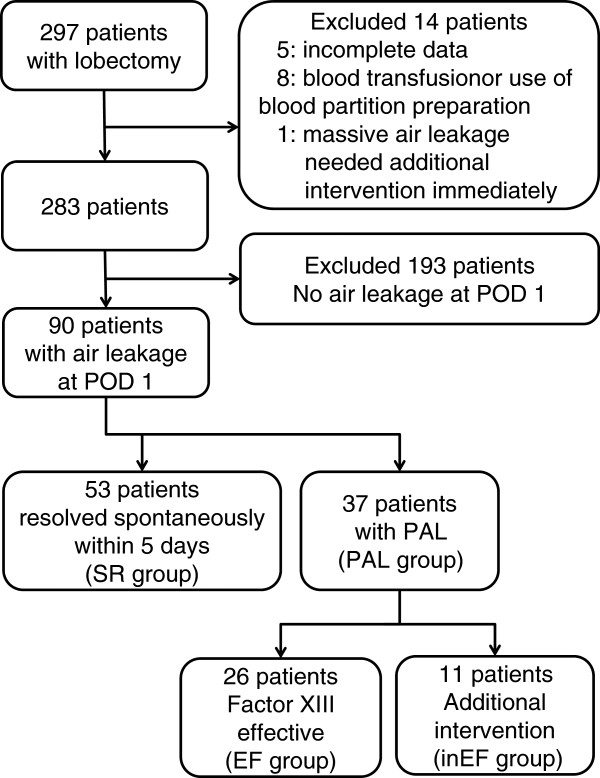
**Flow chart of patient enrollment and classification.**

Of the cohort of 283 patients, 90 (31.8%) had an air leak on the first postoperative day. These patients were divided into three groups: patients were allocated to group SR (Spontaneous Resolution) if the air leak resolved spontaneously within 5 days (n = 53), to group EF (EFfective) if the air leak resolved during the 5-day course of FXIII concentrate (n = 26) or to group inEF (inEFfective) if additional treatment was required to stop the air leak despite administration of FXIII concentrate (n = 11). The PAL group comprised patients in both the EF and inEF groups (n = 37). The allocation of patients to each group is summarized in the schematic shown in Figure 1.

First, the SR and PAL groups were compared to establish the perioperative risk factors for PAL. Thereafter, the EF and inEF groups were compared to find predictors that exogenous FXIII concentrate administration would be beneficial. The independent variables collected were age, sex, BMI, smoking status, presence of comorbid chronic obstructive lung disease (COPD) and interstitial pneumonia (IP) based on the radiological findings of emphysematous or honeycomb changes, details of surgery and the findings of perioperative laboratory investigations. The extent of air leakage during the drainage period was categorized and recorded according to the four qualitative categories developed by Cerfolio and colleagues [20]. We also recorded information about other potential influences such as the history of lung disease and treatments, the presence of vascular disease, hematologic disease or autoimmune disorders, and the use of antiplatelet drugs, anticoagulant drugs or systemic corticosteroids. As these factors were only found to be present in a few patients, these were excluded from the final analysis.

The study was performed in accordance with the Declaration of Helsinki and received approval from the institutional review board of the Osaka City University (Osaka, Japan, reference number 2794) with waiver of informed consent because of its retrospective design. Written informed consent for the use of partitioned plasma products was obtained from all patients before treatment.

### Statistical analysis

Associations between the duration of postoperative air leak and clinical variables were sought using the Mann–Whitney U test and the chi-square test. For the correlation between FXIII and other factors, Pearson product–moment correlation coefficient (Pearson’s r) was calculated. Receiver operating characteristic (ROC) curves were constructed to determine the optimal cut-off point for the effect of exogenous FXIII concentrate to distinguish between the EF and inEF groups. The area under the ROC curve (AUC) was calculated. The threshold of statistical significance was set at p <0.05. All statistical analyses were performed using JMP version 10.0 (SAS Institute, Cary, NC, USA).

## Results

The median age of the 90 patients with air leak on the first postoperative day after pulmonary lobectomy was 67 years, the median BMI was 21.2 kg/m^2^, and the majority (72 patients, 80.0%) was male (Table 1). While the median of their respiratory function tests lay within the normal range, 59% had been diagnosed with COPD or IP clinically or on imaging studies. Upper lobectomy or bilobectomy was undertaken in 58 patients (64.4%), which was a significantly greater proportion than those with no air leak on the first postoperative day (99 of 193 patients, 51.3%; p = 0.038). Pleural adhesions were observed in 55 patients (61.1%), 14 of whom had extensive adhesions (covering more than the surface of one lobe). Intraoperative air leaks were detected and treated in 87 patients (96.7%), nine by spraying fibrin glue, four by suturing alone, two by overlaying a polyglycolic acid sheet with fibrin glue and 24 by suturing followed by fibrin glue spray, but the majority (48 patients, 53% of all, or 55% of 87 patients) were treated using all three approaches. On the first postoperative day, air leak was only detectable with coughing in 36 patients (40%), but 12 (13%) had a continuous leak. Air leak on normal expiration was evident in the remaining 42 patients (47%). The original air leak grading system proposed by Cerfolio and coworkers also defined “inspiratory only” category [20], but there were no patients with such an air leak in our cohort. The median duration of postoperative air leak was 4 days (range, 2–21 days), and the median duration of drainage was 6 days (range, 2–29 days).

**Table 1:** **Characteristics of the 90 patients with air leak on the first postoperative day after pulmonary lobectomy**

Factor	n = 90
Male sex	72 (80%)
Age (years)	67 (44–80)
BMI kg/m^2^	21.2 (14.3–30.6)
Smoking Index	900 (0–3000)
Never-smoker	14 (16%)
Asbestos exposure	18 (20%)
FEV1.0%	71.4 (33.9–89.6)
DLCO%	88.1 (55.1–186.6)
COPD/IP (%)	53 (59%)
Upper or bilobectomy	58 (64%)
Pleural Adhesion	
Major	14 (16%)
Minor	41 (46%)
Duration of air leak (days)	4 (2–21)
Repair air leak during operation	
No	3 (3%)
Fibrin glue only	9 (10%)
Suture only	4 (5%)
PGA sheet and fibrin glue	2 (2%)
Suture and fibrin glue	24 (27%)
Suture, PGA and fibrin glue	48 (53%)
Air leak at first postoperative day	
Forced expiratory only	36 (40%)
Expiratory only	42 (47%)
Continuous	12 (13%)
Duration of air leakage (days)	4 (2–21)
Duration of drainage (days)	6 (2–29)
Drainage amount in first 5 days (ml)	1135 (365–2775)

Results are expressed as median and range.

*Abbreviations:* *BMI* body mass index, *COPD* chronic obstructive pulmonary disease, *DLCO* carbon monoxide diffusing capacity, *FEV1.0%* forced expiratory volume in 1 s (predicted), *IP* interstitial pneumonia, *Pleural adhesion* pleural adhesion covering more than the surface of one lobe was classified as major, and less than the surface of one lobe as minor.

Table 2 shows the demographic and clinical characteristics of the SR and PAL groups. The median durations of air leak and drainage in the SR group were 3 days (interquartile range [IQR], 2–4 days) and 5 days (IQR, 4–6 days), respectively. In the PAL group, the median duration of air leak and drainage were both significantly longer: 7 days (IQR, 6–10 days) and 10 days (IQR, 7–16 days), respectively (p <0.001 for both). The method used to repair intraoperative air leak appeared to influence the risk of PAL: the PAL group contained a significantly greater proportion of patients treated with both a polyglycolic acid sheet and suturing (p = 0.008). There were also significant differences between the SR and PAL groups in terms of the amount of air leak on the first postoperative day and change in plasma FXIII activity. The proportion of patients with continuous air leak on the first postoperative day was significantly lower in the SR group than the PAL group (4% *versus* 27%, respectively; p = 0.006). The SR group also exhibited a significantly greater decrease ratio in plasma FXIII activity on the fifth postoperative day compared with the PAL group (33% *versus* 22%, p = 0.044). There were no significant differences in preoperative or postoperative FXIII activity between the groups.

**Table 2:** **Comparison of the clinical characteristics of patients with or without spontaneous resolution of pulmonary air leak within the first five postoperative days**

Factor	SR	PAL	P value
Sex (Male/Female)	42/11	30/7	0.83
Age	68 (63–72)	69 (64–73)	0.562
BMI	22.1 (19.0–23.9)	20.2 (18.8-23.9)	0.234
Smoking Index	880 (345–1600)	940 (600–1600)	
Never/Ever smoker	9/44	5/32	0.655
Diabetes (Yes/No)	7/46	9/28	0.174
FEV1.0%	72.8 (66.0–79.1)	70.8 (63.0–77.2)	0.325
DLCO%	92.7 (77.0–114.9)	84.4 (70.8–102.4)	0.219
COPD/IP (Yes/No)	29/24	24/13	0.336
Operative procedure (upper or bilobectomy/other)	35/18	23/14	0.706
Pleural Adhesion (major/minor or no)	7/46	7/30	0.462
Repair air leak during operation			
No	9	3	0.008
PGA sheet or suture	23	7	
Both PGA and suture	21	27	
Fibrin glue during operation (Yes/No)	48/5	35/2	0.483
Air leak at first postoperative day			
Forced expiratory only	23	13	0.006
Expiratory only	28	14	
Continuous	2	10	
Duration of air leak (days)	3 (2–4)	7 (6–10)	<0.001
Duration of drainage (days)	5 (4–6)	10 (7–16)	<0.001
Drainage amount in 5 days (ml)	1105 (849–1485)	1207 (1014–1542)	0.493
Plasma FXIII activity (%)			
Before operation	104 (86–135)	111 (95–132)	0.678
Postoperative day 5	74 (60–87)	78 (68–103)	0.124
Decrease ratio (%)	33 (18–45)	22 (7–36)	0.044

Results are expressed as median and interquartile range.

*Abbreviations: FXIII* factor XIII, *SR* spontaneous resolution group, *PAL* prolonged air leak (>5 days) group, *BMI* body mass index, *PGA* polyglycolic acid.

Table 3 shows the clinical characteristics of the EF and inEF groups. We did not detect any side effects or adverse events attributable to the administration of exogenous factor XIII concentrate. Patients in the inEF group were significantly older (68 years *versus* 72 years, p = 0.018) and had lower BMI (p <0.001) than the EF group. Interestingly, the postoperative plasma FXIII activity of patients in the EF group was significantly lower than those in the inEF group (74% *versus* 91%, p = 0.030), but not significantly different from those in the SR group (Table 2). There was no significant difference in the perioperative change in plasma FXIII activity between the EF and inEF groups (24% *versus* 14%, p = 0.393), or between the SR and EF groups (33% *versus* 24%, p = 0.171); however, there was a significant difference in the perioperative change in FXIII between the SR and inEF groups (33% *versus* 14%, p = 0.048). Patients’ age, BMI and postoperative FXIII activity showed either no (FXIII and age: Pearson product–moment correlation coefficient = 0.03) or weak (FXIII and BMI: correlation coefficient = −0.24) correlations.

**Figure 2:**
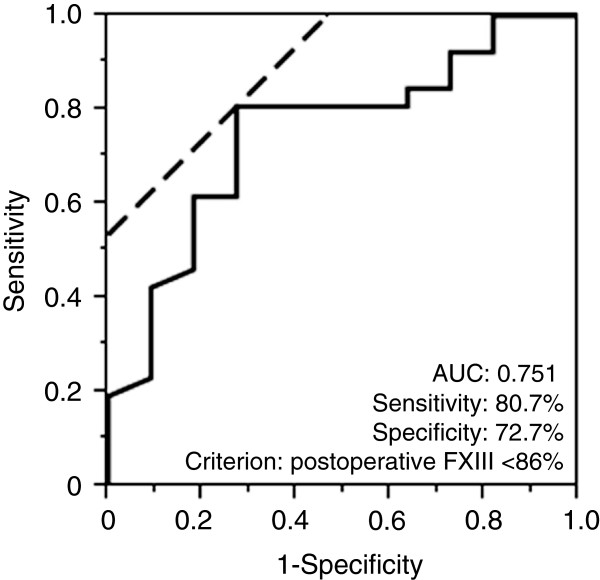
**Receiver operating characteristic (ROC) curve for postoperative plasma factor XIII activity in the EF and inEF groups.** Area under the curve (AUC) = 0.751. The calculated optimal cut-off point for factor XIII activity on the fifth postoperative day to distinguish effectiveness of exogenous factor XIII concentrate administration was 86% (Youden Index = 0.535); the overall sensitivity was 80.7% and the overall specificity was 72.7%.

Finally, we analyzed the ROC curve to estimate the optimal cut-off value of postoperative plasma factor XIII activity to identify patients likely to respond to treatment with exogenous FXIII concentrate. The area under the ROC curve for postoperative FXIII activity was 0.751 (Figure 2). The optimal cut-off point for postoperative FXIII activity was 86%, at which the overall sensitivity for predicting resolution of air leak during FXIII therapy was 80.7% (21 of the 26 patients in whom air leak resolved during FXIII therapy) and the overall specificity was 72.7% (eight of 11 patients in whom air leak persisted despite FXIII therapy).

**Table 3:** **Comparison of clinical characteristics of patients in whom factor XIII concentrate was effective for persistent air leak and those in whom it was ineffective**

Variables	EF	inEF	P value
Sex (Male/Female)	21/5	9/2	0.941
Age	68 (61–72)	72 (69–77)	0.018
BMI	21.4 (19.9–24.9)	18.7 (18.0–19.4)	<0.001
Smoking Index	950 (600–1783)	940 (600–1400)	
Ever-smoker/Never	22/4	10/1	0.609
Diabetes (Yes/No)	6/20	3/8	0.786
FEV1.0%	71.1 (59.6–77.2)	70.0 (64.9–83)	0.561
DLCO%	84.4 (72.4–104.1)	82.3 (68.6–99.9)	0.698
COPD/IP (Yes/ No)	15/11	9/2	0.160
Operative procedure (upper or bilobectomy/other)	17/9	6/5	0.534
Pleural Adhesion (major/minor or no)	6/20	1/10	0.321
Repair air leak during operation			
No	3	0	0.137
PGA sheet or suture	3	4	
Both PGA and suture	20	7	
Fibrin glue during operation (Yes/No)	25/1	10/1	0.519
Air leak at first postoperative day			
Forced expiratory only	8	5	0.276
Expiratory only	12	2	
Continuous	6	4	
Duration of air leak (days)	7 (6–9)	15 (10–19)	<0.001
Duration of drainage (days)	8 (7–11)	17 (15–22)	<0.001
Drainage amount of 5 days (ml)	1220 (1050–1543)	1054 (626–1554)	0.291
Plasma factor XIII activity (%)			
Before operation	110 (89–130)	123 (99–147)	0.394
Postoperative day 5	74 (64–86)	91 (77–125)	0.030
Decrease ratio (%)	24 (7–39)	14 (1–36)	0.393

Results are expressed as median and interquartile range.

*Abbreviations: EF* group of patients in whom the air leak resolved during FXIII administration, *inEF* group of patients who required additional intervention despite FXIII, *BMI* body mass index, *COPD* chronic obstructive pulmonary disease, *DLCO* carbon monoxide diffusing capacity, *FEV1.0%* forced expiratory volume in 1 s (predicted), *IP* interstitial pneumonia, *PGA* polyglycolic acid.

## Discussion

Prolonged air leak is a common complication of pulmonary resection that can have serious consequences, and has the potential to prolong hospital stay [2]. Several techniques and therapies have been developed to address PAL, such as the pleural tent technique [21], the use of surgical sealants [22] and endobronchial valves [23], and different chest drainage management strategies [24]. Nonetheless, the choices remain relatively limited and their results inconsistent, apart from the more invasive procedures.

In our cohort, three significant risk factors for PAL were detected: a reduced proportional reduction in the activity of FXIII, which likely reflects impaired consumption of FXIII; the method used to repair any intraoperative air leak detected; and the amount of air leak on the first postoperative day, which also reflected the outcome of efforts to effect an intraoperative repair. Risk factors reported by other investigators, such as age, BMI or FEV [5–7], did not appear to play a role in our cohort, but this may have arisen as a result of selection bias. The subjects of previous studies included patients with no air leak after surgery, who were excluded from our analysis. This would likely have eliminated the risk factors for *developing* an air leak, allowing us to assess the influence of a variety of perioperative factors on the *maintenance* of a leak and thereby those contributing to a delay in the healing of a pulmonary fistula. As the perioperative change in plasma FXIII activity was the only factor that appeared to influence PAL resolution in our cohort, it may play a role in the prompt closure of postoperative air leaks.

Blood coagulation FXIII was initially identified as a component of the hemostatic system [25]. It exists in plasma as a heterotetramer consisting of two enzymatic A-subunits synthesized by cells of bone marrow origin, and two carrier/inhibitor B-subunits synthesized by hepatocytes. In the final stage of the coagulation cascade, circulating FXIII is bound to fibrinogen and activated by thrombin and calcium ions. Activated FXIII, which is a transformed A-subunit dimer, then cross-links fibrin chains and stabilizes them. Recently, it has become clear that FXIII is also involved in wound healing. Studies of FXIII-deficient animals [26] and patients with acquired or inherited FXIII deficiency [27–30] reported that the level of plasma FXIII activity influences the time taken for wound healing. Cario et al. found that FXIII significantly enhanced epithelial restitution *in vitro* in a TGF-β-independent manner [17], and Inbal et al. showed that impaired wound healing could be restored by replenishing plasma FXIII with human FXIII concentrate in a mouse experimental model [26].

We compared the EF group and inEF group to examine the effect of exogenous FXIII concentrate administration. The EF group was significantly younger, had higher BMI and had significantly lower postoperative plasma FXIII activity compared with the inEF group. As there were no significant correlations between postoperative FXIII activity, age and BMI, each of these factors may have an independent effect on the outcome of administration of exogenous FXIII concentrate. This is the first occasion on which these perioperative changes in FXIII activity have been described: the SR group had the lowest postoperative FXIII activity (74%) and the largest proportional perioperative decrease in FXIII activity (33%) of all groups. Postoperative FXIII activity was not statistically different in the EF group, but the perioperative decrease was only 24%. Interestingly, postoperative FXIII activity was highest (91%) in the inEF group, and activity had only fallen by 14% by the fifth postoperative day.

Our findings suggest that there could be two separate mechanisms underpinning PAL. First, PAL may be a consequence of insufficient plasma FXIII activity, and may therefore respond to treatment with exogenous FXIII concentrate. This type of PAL would be characterized by low postoperative FXIII activity, which would be reflected by a relatively low proportional change in activity compared with patients in whom air leak resolves spontaneously. In support of this hypothesis, we found that PAL resolved in 85% of patients within 3 days of starting exogenous FXIII concentrate treatment (data not shown) in the EF group. The second type of PAL could be caused by a lack of consumption of FXIII, the postoperative activity of which is therefore higher and the proportional perioperative decrease lower. We cannot, however, draw any conclusions about why FXIII consumption would be disordered in these patients from the results of this study. As patients in the inEF group ultimately needed invasive therapies, such as pleural adhesion therapy to promote wound healing cascade, or secondary surgery to physically close the wound, a potential explanation is that the blood coagulation and wound healing cascades had become exhausted and consumption of factor XIII had ceased before the resolution of air leak. For this type of PAL, administration of additional exogenous FXIII concentrate might not be effective.

We found that the optimal cut-off point of postoperative plasma FXIII activity that differentiated between the EF and inEF groups most effectively was 86%, compared with a cut-off of 70% reported in a previous study [31]. Given the limitations of our study design and the relatively small sample size, we recommend that other clinical factors are also taken into account when determining treatment strategies for patients with PAL in addition to the use of factor XIII concentrate.

## Conclusions

We found that plasma FXIII appears to play an important role in closing pulmonary fistulae in patients with PAL after pulmonary lobectomy for non-small cell lung cancer. In 85% of the EF group, air leaks resolved within 3 days of commencing exogenous FXIII concentrate treatment, and no apparent adverse events were observed. Interestingly, plasma FXIII activity fell less during the course of treatment in those in whom PAL did not resolve, suggesting that lack of consumption or utilization of endogenous FXIII may delay healing of pulmonary fistulae. As a non-invasive treatment, this strategy is a particularly useful and safe therapeutic option for those who might not tolerate more invasive therapies. Nonetheless, as our study design was observational and retrospective, and was conducted in a single center, further randomized controlled prospective studies with larger numbers of subjects will be needed to validate our findings.

## Abbreviations

BMI: Body mass index
COPD: Chronic obstructive pulmonary disease
DLCO: Carbon monoxide diffusing capacity
EF group: Group of patients with an air leak that resolved during FXIII administration
FEV_1_: Forced expiratory volume in the first second
FXIII: Factor XIII
inEF group: Group of patients who required additional therapies
IP: Interstitial pneumonia
IQR: Interquartile range
SR group: Group in which air leak resolved spontaneously
PAL: Prolonged air leak (more than 5 days)
PAL group: Group of patients with prolonged air leak
ROC: Receiver operating characteristic.
